# Erratum: Theory of Thomson scattering in inhomogeneous media

**DOI:** 10.1038/srep26366

**Published:** 2016-06-27

**Authors:** P. M. Kozlowski, B. J. B. Crowley, D. O. Gericke, S. P. Regan, G. Gregori

Scientific Reports 6: Article number: 2428310.1038/srep24283; published online: 04122016; updated: 06272016.

This Article contains errors in the order of the References. The correct Reference list appears below.

1. Froula, D., Glenzer, S. H., Luhmann, N. C. & Sheffield, J. In *Plasma scattering of electro-magnetic radiation* 2nd edn, Ch. 11, 309–334 (Academic Press, 2011).
2. Pile, D. X-rays: First light from SACLA. *Nat. Photon*. **5**, 456–457 (2011).
3. Emma, P. *et al*. First lasing and operation of ångstrom-wavelength free-electron laser *Nat. Photon*. **4**, 641–647 (2010).
4. Glenzer, S. H. & Redmer, R. X-Ray Thomson scattering in high energy density plasmas. *Rev. Mod. Phys*. **81**, 1625–1663 (2009).
5. Evans, D. E. & Katzenstein, J. Laser light scattering in laboratory plasmas. *Rep. Progr. Phys*. **32**, 207–271 (1969).
6. Gregori, G., Glenzer, S. H., Rozmus, W., Lee, R. W. & Landen, O. L. Theoretical model of x-ray scattering as a dense matter probe. *Phys. Rev*. *E* **67**, 026412 (2003).
7. Glenzer, S. H. *et al*. Demonstration of Spectrally Resolved X-Ray Scattering in Dense Plasmas. *Phys. Rev. Lett*. **90**, 175002 (2003).
8. Glenzer, S. H. *et al*. Observations of Plasmons in Warm Dense Matter. *Phys. Rev. Lett*. **98**, 065002 (2007).
9. Garcia Saiz, E. *et al*. Probing warm dense lithium by inelastic x-ray scattering. *Nature Phys*. **4**, 940–944 (2008).
10. Kritcher, A. L. *et al*. Ultrafast X-ray Thomson Scattering of Shock-Compressed Matter. *Science* **322**, 69–71 (2008).
11. Brown, C. R. D. *et al*. Evidence for a glassy state in strongly driven carbon. *Sci. Reports* **4**, 5214 (2014).
12. Chapman, D. A. *et al*. Observation of Finite-Wavelength Screening in High-Energy-Density Matter. *Nature Comm*. **6**, 6839 (2015).
13. Peacock, N. J., Robinson, D. C., Forrest, M. J., Wilcock, P. D. & Sannikov, V. V. Measurement of the electron temperature by Thomson scattering in tokamak T3. *Nature* **224**, 488–490 (1969).
14. Forrest, M. J., Carolan, P. G. & Peacock, N. J. Measurement of the magnetic field in a tokamak using laser light scattering. *Nature* **271**, 718–722 (1978).
15. Nagler, B. *et al*. Turning solid aluminium transparent by intense soft X-ray photoionization. *Nature Phys*. **5**, 693–696 (2009).
16. Emma, P. *et al*. First lasing and operation of ångstrom-wavelength free-electron laser *Nat. Photon*. **4**, 641–647 (2010).
17. Drake, R. P. In *High energy density physics*, Ch. 11, 461–464 (Springer, 2006).
18. Hurricane, O. A. *et al*. Fuel gain exceeding unity in an inertially confined fusion implosion *Nature* **506**, 343–348 (2014).
19. Regan, S. P. *et al*. Inelastic X-Ray Scattering from Shocked Liquid Deuterium. *Phys. Rev. Lett*. **109**, 265003 (2012).
20. Gregori, G. *et al*. Thomson scattering measurements in atmospheric plasma jets. *Phys. Rev. E*. **59**, 2286 (1999).
21. Ichimaru, S. In *Basic Principles of Plasma Physics*, Ch. 7 Fluctuations, 251–255 Addison Wesley (1973).
22. Crowley, B. J. B. & Gregori, G. X-ray scattering by many-particle systems. *New J. Phys*. **15**, 015014 (2013).
23. Kittel, C. In *Introduction to solid state physics* 8th edn, Ch. 2, 23–45 (Wiley, 2004).
24. Chihara, J. Difference in X-ray scattering between metallic and non-metallic liquids due to conduction electrons. *Phys. F:Met. Phys*. **17**, 295 (1987).
25. Kadanoff, L. P. & Baym, G. *Quantum Statistical Mechanics*. W.A. Benjamin Inc. (1962).
26. Kwong, N.-H. & Bonitz, M. Real-time Kadano-Baym approach to plasma oscillations in a correlated electron gas. *Phys. Rev. Lett*. **84**, 1768 (2000).
27. Gregori, G., Glenzer, S. H. & Landen, O. L. Generalized x-ray scattering cross section from nonequilibrium plasmas. *Phys. Rev. E*. **74**, 026402 (2006).
28. Gregori, G. *et al*. Derivation of the static structure factor in strongly coupled non-equilibrium plasmas for X-ray scattering studies. *High Energy Density Physics* **3**, 99–108 (2007).
29. Belyi, V. V. Fluctuation-Dissipation Relations for a Nonlocal Plasma*. Phys. Rev. Lett*. **88**, 255001 (2002).
30. Bornatici, M. & Kravtsov, A. Yu. Comparative analysis of two formulations of geometrical optics. The effective dielectric tensor. *Plasma Phys. Controlled Fusion* **42**, 255 (2000).
31. Pitaevskii, L. P. & Lifshitz, E. M. *Physical Kinetics*. Butterworth-Heinemann (1981).
32. Pines, D. & Nozieres, P. In *The Theory of Quantum Liquids*, Ch. 2, 130–136 (Westview Press, 1999).
33. Döppner, T. *et al*. Temperature measurement through detailed balance in x-ray Thomson scattering. *High Energy Dens. Phys*. **5**, 182–186 (2009).
34. Fäustlin, R. R. *et al*. Observation of Ultrafast Nonequilibrium Collective Dynamics in Warm Dense Hydrogen. *Phys. Rev. Lett*. **104**, 125002 (2010).
35. Chapman, D. A. & Gericke, D. O. Analysis of Thomson Scattering from Nonequilibrium Plasmas. *Phys. Rev. Lett*. **107**, 165004 (2011).
36. Glenzer, S. H. *et al*. Thomson Scattering from High-Z Laser-Produced Plasmas*. Phys. Rev. Lett*. **82**, 97–100 (1999).
37. Rozmus, W., Glenzer, S. H., Estabrook, K. G., Baldis, H. A. & MacGowan, B. J. Modeling of Thomson Scattering Spectra in High-Z, Laser-produced Plasmas. *Astrophys. J. Suppl. S*. **127**, 459–463 (2000).
38. Falk, K. *et al*. Comparison between x-ray scattering and velocity-interferometry measurementsfrom shocked liquid deuterium. *Phys. Rev. E* **87**, 043112 (2013).
39. Chapman, D. A. *et al*. Simulating x-ray Thomson scattering signals from high-density, millimetre-scale plasmas at the National Ignition Facility *Phys. Plasmas* **21**, 082709 (2014).
40. Sperling, P., Liseykina, T., Bauer, T. & Redmer, R. Time-resolved Thomson scattering on high-intensity laser-produced hot dense helium plasmas *New J. Phys*. **15**, 025041 (2013).
41. Buhmann, S. Y., Butcher, D. T. & Scheel, S. Macroscopic quantum electrodynamics in nonlocal and nonreciprocal media. *New J. Phys*. **14**, 083034 (2012).

In addition, there is a typographical error in the Introduction section,

“This limitation becomes particularly restrictive when considering the ultra-short x-ray pulses and near diffraction limited laser spot sizes of fourth generation light sources^15,16^”.

should read:

“This limitation becomes particularly restrictive when considering the ultra-short x-ray pulses and near diffraction limited laser spot sizes of fourth generation light sources^3,15^”.

